# The REVAMP study: research exploring various aspects and mechanisms in preeclampsia: study protocol

**DOI:** 10.1186/s12884-019-2450-0

**Published:** 2019-08-23

**Authors:** Nisha S. Wadhwani, Deepali P. Sundrani, Girija N. Wagh, Savita S. Mehendale, Manish M. Tipnis, Priscilla C. Joshi, Arun S. Kinare, Sanjay K. Lalwani, Narayanan S. Mani, Nomita Chandhiok, Giriraj R. Chandak, Sanjay A. Gupte, Caroline H. D. Fall, Sadhana R. Joshi

**Affiliations:** 1 Mother and Child Health, Interactive Research School for Health Affairs (IRSHA), Bharati Vidyapeeth (Deemed to be University), Pune Satara Road, Pune, 411043 India; 2 Department of Obstetrics and Gynaecology, Bharati Medical College and Hospital, Bharati Vidyapeeth (Deemed to be University), Pune, 411043 India; 3 Gupte Hospital and Research Centre, Pune, 411004 India; 4 Department of Radiodiagnosis, Bharati Medical College and Hospital, Bharati Vidyapeeth (Deemed to be University), Pune, 411043 India; 5 Department of Pediatrics, Bharati Medical College and Hospital, Bharati Vidyapeeth (Deemed to be University), Pune, 411043 India; 6 Department of Pathology, Bharati Medical College and Hospital, Bharati Vidyapeeth (Deemed to be University), Pune, 411043 India; 7 Division of Reproductive Biology, Maternal and Child Health, Indian Council of Medical Research (ICMR), New Delhi, 110029 India; 8 Genomic Research on Complex diseases (GRC Group), Council of Scientific and Industrial Research -Centre for Cellular and Molecular Biology, (CSIR-CCMB), Hyderabad, 500007 India; 9 MRC Lifecourse Epidemiology Unit, University of Southampton, Southampton, UK

**Keywords:** Preeclampsia, Long chain polyunsaturated fatty acids, Developmental programming

## Abstract

**Background:**

Preeclampsia is a major cause of maternal, fetal and neonatal morbidity and mortality, particularly in developing countries. Considering the burden of preeclampsia and its associated complications, it is important to understand the underlying risk factors and mechanisms involved in its etiology. There is considerable interest in the potential for dietary long chain polyunsaturated fatty acids (LCPUFA) as a therapeutic intervention to prevent preeclampsia, as they are involved in angiogenesis, oxidative stress, and inflammatory pathways.

**Methods:**

The REVAMP study (Research Exploring Various Aspects and Mechanisms in Preeclampsia) follows a cohort of pregnant women from early pregnancy until delivery to examine longitudinally the associations of maternal LCPUFA with clinical outcome in preeclampsia. A multisite centre for advanced research was established and pregnant women coming to Bharati hospital and Gupte hospital, Pune, India for their first antenatal visit are recruited and followed up at 11–14 weeks, 18–22 weeks, 26–28 weeks, and at delivery. Their personal, obstetric, clinical, and family history are recorded. Anthropometric measures (height, weight), food frequency questionnaire (FFQ), physical activity, socioeconomic status, fetal ultrasonography, and color Doppler measures are recorded at different time points across gestation. Maternal blood at all time points, cord blood, and placenta at delivery are collected, processed and stored at − 80 °C. The children’s anthropometry is assessed serially up to the age of 2 years, when their neurodevelopmental scores will be assessed.

**Discussion:**

This study will help in early identification of pregnant women who are at risk of developing preeclampsia. The prospective design of the study for the first time will establish the role of LCPUFA in understanding the underlying biochemical and molecular mechanisms involved in preeclampsia and their association with developmental programming in children.

## Background

Preeclampsia, the most common hypertensive disorder of pregnancy is characterized by new-onset hypertension and proteinuria in pregnancy [1]. The global incidence, ranges between 2 and 10% [2] and is reported to be 8–10% in India [3]. In developing countries, around 40–60% of maternal deaths occur due to preeclampsia [4]. It has therefore been identified as a priority area in developing countries as per the Millennium Development Goal to reduce maternal mortality [5]. There is considerable evidence that women with preeclampsia are at an increased risk of developing cardiovascular disease in later life [6]. Furthermore, children born to mothers with preeclampsia are at an increased risk for developing cardiovascular disease and impaired cognitive function [7, 8]. In view of the enormity of the consequences associated with preeclampsia it is vital to understand its risk factors and underlying mechanisms that would enable appropriate intervention at specific gestational stage or earlier.

Preeclampsia is known to be associated with abnormal placentation, characterized by shallow trophoblast invasion and narrow spiral arteries, which lead to hypoxia and endothelial dysfunction [9, 10]. It is known that good maternal nutrition is essential for normal placental development. Any disruption during the course of implantation and development of the placenta affects its functioning and adversely influences fetal growth [11].

Long chain polyunsaturated fatty acids (LCPUFA) and their metabolites play a role in implantation and are required at various stages of placental development and function [12, 13]. They are involved in processes associated with placental growth and development, notably angiogenesis, oxidative stress, and inflammation [14]. LCPUFA regulate the expression of growth factors involved in placental development by modulating the activity of transcription factors like peroxisome proliferator-activated receptor (PPAR) and hypoxia inducible factor-1 (HIF-1) [15].

LCPUFA such as arachidonic acid (AA) and docosahexaenoic acid (DHA) are essential for fetal growth as well as the development of the brain and retina [16, 17]. However, due to the inability of the fetus to synthesize these LCPUFA, it largely depends on the maternal supply of LCPUFA [18]. Phospholipids constitute a major fraction involved in the transfer of LCPUFA from mother to fetus across the placenta [19] and are linked to LCPUFA such as DHA through the one carbon metabolism [20].

One carbon metabolism includes a set of reactions that are involved in the addition, transfer or removal of one carbon units in cellular metabolic pathways [21]. Folate plays a key role in the one carbon cycle where it donates one carbon in the form of 5-methyltetrahydrofolate for the remethylation of homocysteine to methionine which is catalyzed by the vitamin B_12_–dependent enzyme, methionine synthase [22–24]. Methionine, an essential amino acid is subsequently converted to S-adenosylmethionine (SAM) which is the universal methyl donor required for the methylation of various cellular methyltransferase reactions such as methylation of phospholipids, nucleic acids, and neurotransmitters [25, 26]. Phospholipids are the major methyl acceptors in the one carbon cycle. Phosphatidylethanolamine-N-methyltransferase (PEMT) utilizes SAM for the methylation of phosphatidylethanolamine (PE) to form phosphatidylcholine (PC) [27]. PEMT is considered to play an important role in the transfer of LCPUFA like DHA from liver to the plasma and other tissues [28–30].

The role of one carbon metabolism in developmental programming of chronic diseases has been emphasized with reference to its important role in DNA methylation, a crucial epigenetic regulatory mechanism [31, 32]. Altered developmental programming is known to occur when there is improper establishment of epigenetic modifications (e.g. DNA and histone methylation) [33]. In several studies, we have demonstrated that changes in maternal levels of LCPUFA and one carbon cycle metabolites influences DNA methylation, resulting in altered expression of genes involved in placental growth and development [20, 34–36].

It is well known that preeclampsia is closely associated with placental dysfunction characterized by altered maternal and placental levels of angiogenic factors like vascular endothelial growth factor (VEGF) and placental growth factor (PlGF) and its receptors fms-like tyrosine kinase-1 (Flt-1) [37–41]. Evidence suggests that the pathogenesis of preeclampsia is initiated by placental ischemia, followed by release of placental anti-angiogenic factors like soluble Flt-1 (sFlt-1) and soluble endoglin (sEng) into the circulation [42].

In earlier cross-sectional studies in women with preeclampsia, we have demonstrated lower levels of maternal DHA, higher homocysteine and oxidative stress; lower angiogenic factors and higher anti-angiogenic factors [43–47]. These findings were observed after the onset of clinical preeclampsia. In order to determine whether these changes are causal, it is important to examine these metabolite levels prospectively from early pregnancy. In a preliminary study involving women with preeclampsia, we observed higher levels of homocysteine, oxidative stress, and sFlt-1/PlGF ratio along with lower levels of maternal DHA as early as 16–20 weeks of gestation [48–52]. There is a need to validate these changes in a larger sample. Further, there is a need to understand the influence of maternal nutritional status, physical activity, and socioeconomic status on the levels of these circulating biomolecules. A combination of biochemical and molecular measures, along with clinical information, especially ultrasonography (USG) and color Doppler measures, will help identify women at risk of developing preeclampsia. Since one carbon cycle metabolites are known to influence placental methylation patterns, it is important to understand the influence of early life exposures on placental epigenetic changes and fetal programming.

With this background, the present longitudinal study aims to follow pregnant women from early pregnancy until delivery, to examine changes across gestation in nutritional, biochemical, and molecular measures and identify the underlying mechanisms which influence the pathophysiology of preeclampsia. The study will also follow up the children’s growth during infancy and their neurodevelopment at the age of 2 years.

## Methods/design

### Hypothesis

We hypothesize that LCPUFA and one carbon metabolite status among Indian women play a causal role in the etiology of preeclampsia, mediated by changes in oxidative stress and growth factors which alter placental development.

### Proposed mechanism

**Fig. 1:**
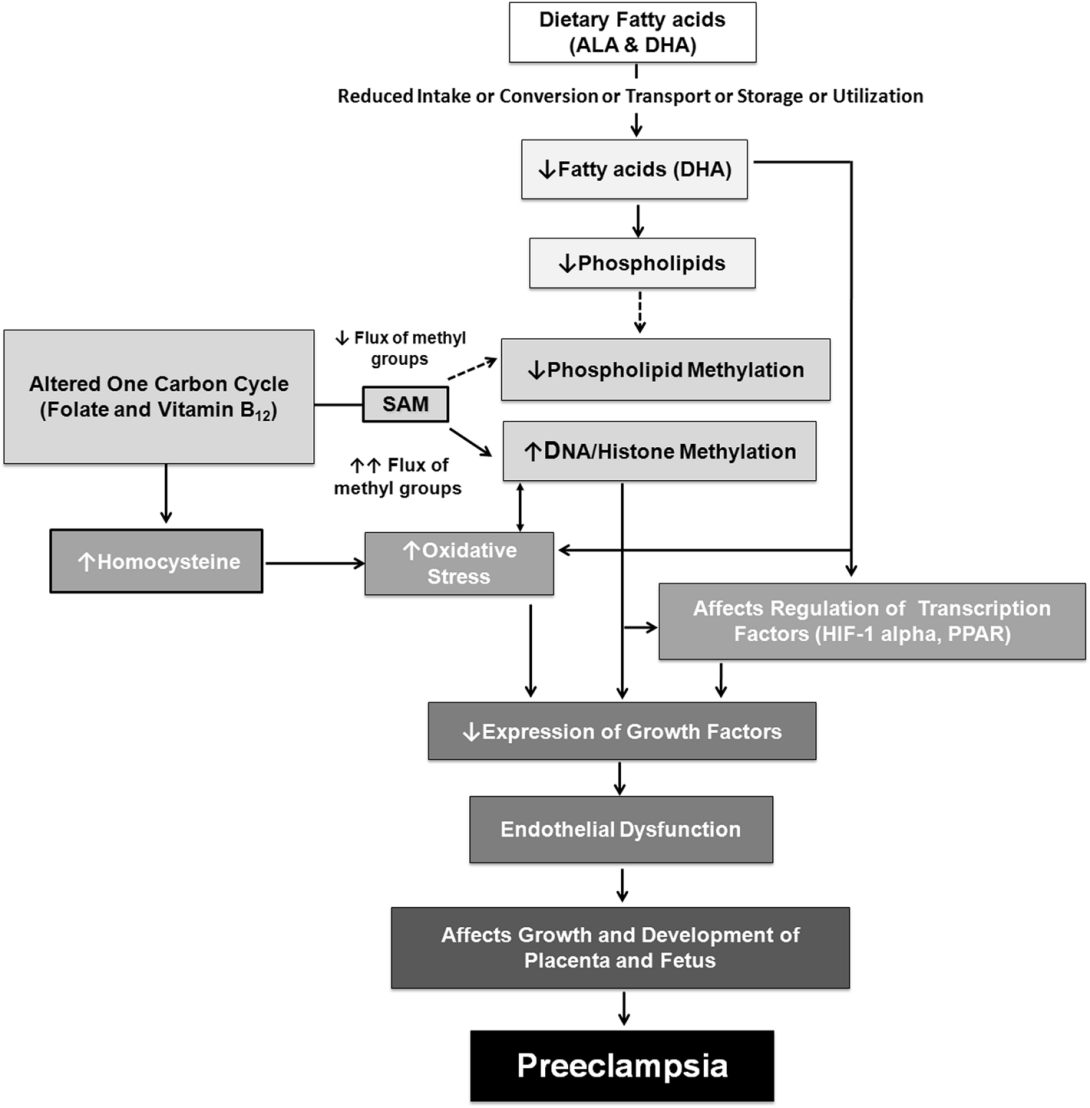
Proposed Mechanism of Altered Maternal Nutrition Leading to Preeclampsia. ALA: alpha linolenic acid; DHA: docosahexaenoic acid; DNA: deoxyribonucleic acid; HIF-1 alpha: hypoxia inducible factor – 1 alpha; PPAR: peroxisome proliferator-activated receptor; SAM: S-adenosylmethionine

Dietary folate and vitamin B_12_ are considered as important nutritional constituents of the one carbon cycle for the supply of methyl groups for the remethylation of homocysteine to methionine which is then converted to SAM, a major methyl donor. Any alteration in the one carbon cycle leads to increased homocysteine levels which is associated with higher oxidative stress. Our studies have demonstrated that folate and vitamin B_12_ are interlinked with DHA in the one carbon cycle. Alpha linolenic acid (ALA), an omega-3 fatty acid is the dietary precursor for the synthesis of LCPUFA such as DHA. Omega-3 fatty acids are extremely important during pregnancy as they are critical building blocks of fetal brain and retina. Any alteration in the conversion, transport, storage or utilization of fatty acids may reduce maternal LCPUFA levels, thereby reducing the levels of placental phospholipids containing these LCPUFA. Membrane phospholipids are the major methyl group acceptors, where the PE containing LCPUFA accepts methyl group from SAM to form PC containing LCPUFA. Reduced phospholipids may therefore divert the flux of methyl groups from SAM to DNA and histone thereby affecting DNA and histone methylation patterns. This may affect the gene expression patterns of various transcription factors and growth factors. In addition, reduced fatty acids are also known to affect the regulation of various transcription factors thereby affecting the expression of various growth factors involved in placental growth and development. This may lead to endothelial dysfunction thereby affecting the growth and development of the placenta and fetus, ultimately resulting into preeclampsia (Fig. 1).

### Objectives

The REVAMP study (Research Exploring Various Aspects and Mechanisms in Preeclampsia) proposes to examine the associations of maternal LCPUFA and one carbon micronutrient status in early gestation with clinical outcome in preeclampsia and to understand the underlying key biochemical and molecular mechanisms. It consists of 4 sub-projects:

#### ***Project 1:*** Longitudinal changes in metabolites of the one carbon cycle in preeclampsia

The study will examine baseline levels (in early pregnancy) at 11–14 weeks of gestation (visit 1 –V1) and changes across gestation [18–22 weeks (visit 2 – V2), 26–28 weeks (visit 3 – V3) and at delivery] in components of the one carbon cycle [(folate, vitamin B_12_, methionine, glutathione, homocysteine, SAM, S-adenosylhomocysteine (SAH)] from early pregnancy until delivery, and placental global DNA methylation levels. It will also examine the relationship of altered one carbon metabolism to methylation potential (SAM: SAH ratio) in the placenta. Furthermore, these changes in the one carbon cycle metabolites will be associated with dietary and lifestyle factors, USG and color Doppler measures.

#### ***Project 2:*** Role of oxidative stress and metabolites of LCPUFA metabolic pathway in preeclampsia

This study will examine levels of oxidative stress markers (protein carbonyl, 8- hydroxyl (OH) guanidine, malondialdehyde (MDA); LCPUFA status and their bioactive metabolites (thromboxane B2; prostaglandin E2) at baseline and across gestation. In addition, measurements of placental phospholipids and gene expression and DNA methylation patterns of PPAR gamma, PEMT, and DNA methyl transferase (DNMT) genes from the placenta will be undertaken.

#### ***Project 3:*** Growth factors and regulation of their gene expression in preeclampsia

This study will analyze levels of angiogenic growth factors – [VEGF, PlGF, sFlt-1, sEng], HIF-1, neurotrophins – [brain derived neurotrophic factor (BDNF), nerve growth factor (NGF) and their receptors] across gestation. It will also examine the associations between altered expression of growth factors involved in placental development, their regulation by methylation as a response to altered one carbon metabolism, and placental histopathological changes. These molecular as well as structural changes in the placenta will further be correlated with clinical parameters for better understanding the etiopathology of preeclampsia.

#### ***Project 4:*** Association of maternal nutrition, biochemical and epigenetic changes with anthropometry and developmental changes in children born to mothers with preeclampsia

**Fig. 2:**
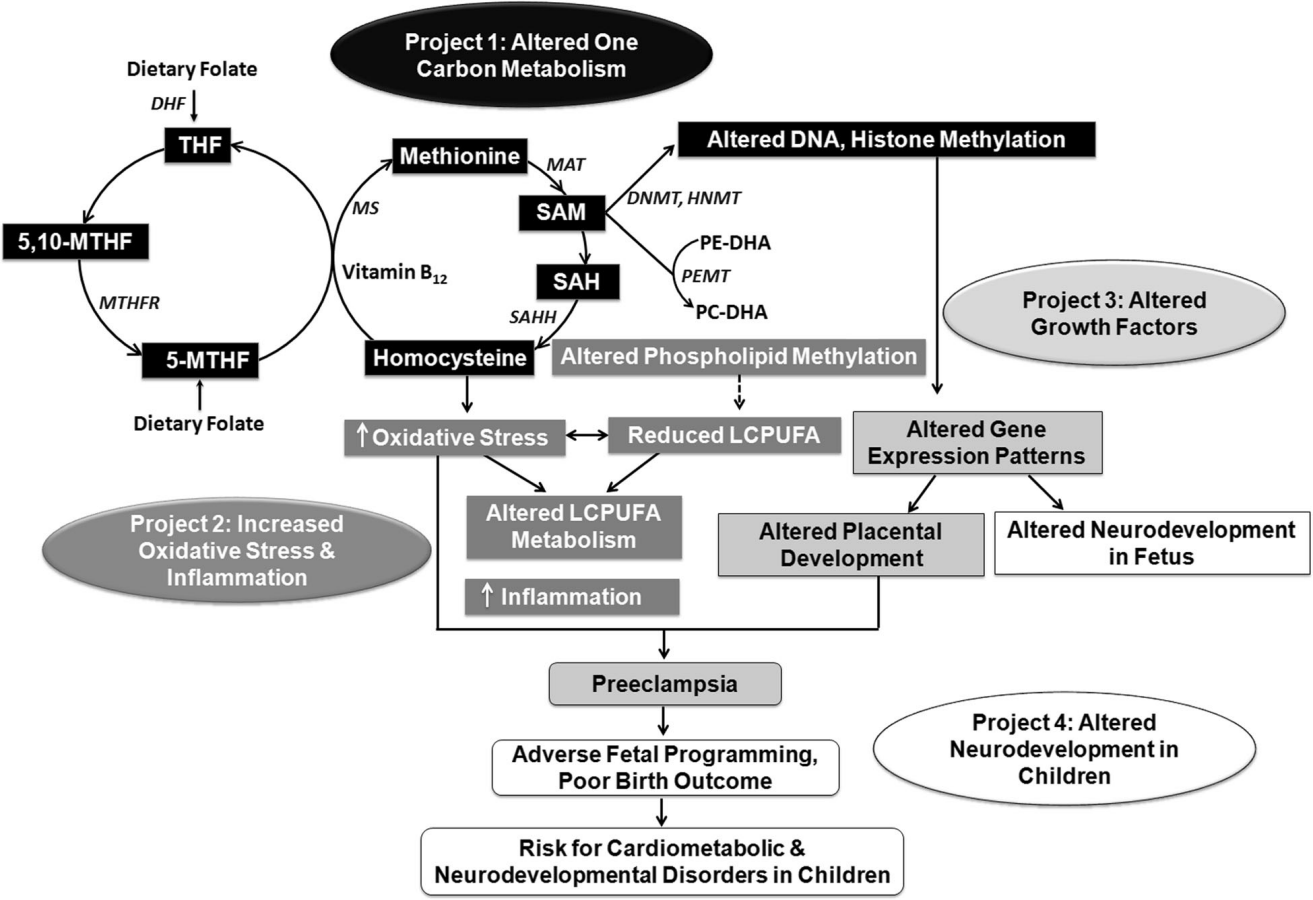
Connectivity between Various Objectives. 5,10-MTHF, 5,10-methylene tetrahydrofolate; 5-MTHF, 5-methylene tetrahydrofolate; DHF, dihydrofolate; DNA, deoxyribonucleic acid, DNMT, DNA methyl transferase; HMT, histone methyl transferase; MAT, methionine adenosyltransferase; MS, methionine synthase; MTHFR, methylene tetrahydrofolate reductase; PC-DHA, phosphatidylcholine with docosahexaenoic acid attached to position 2; PE-DHA, phosphatidylethanolamine with docosahexaenoic acid attached to position 2; PEMT, phosphatidylethanolamine methyl transferase; SAH, S-adenosylhomocysteine; SAHH, S-adenosylhomocysteine hydrolase; SAM, S-adenosylmethionine; THF, tetrahydrofolate

This study aims to follow-up the babies for their postnatal anthropometric measurements at birth, 6, 10, and 14 weeks, 6, 9, 12, 15, 18, and 24 months and developmental scales at 2 years of age. The associations of maternal nutrition, biochemical and epigenetic changes, USG and Doppler measures with anthropometry and developmental scores in children will also be undertaken to better understand the mechanisms involved in the fetal programming of developmental disorders in children born to mothers with preeclampsia. Figure 2 depicts the connectivity between the various objectives.

### Key questions

The proposed study will answer the following questions:

- Do levels of LCPUFA and their metabolites, and one carbon nutrients, differ in early pregnancy between women who do and do not go on to develop preeclampsia?
- Is there evidence that these differences are dietary in origin?
- Do the molecular and biochemical analyzes provide a plausible mechanistic pathway to explain the pathophysiology of preeclampsia?
- Do the early pregnancy biomarkers identified provide a clinically useful prediction of later preeclampsia?
- Does the postnatal growth and cognitive development of children of women with preeclampsia differ from that of controls?
- Are these differences in the children explained by changes in growth factors?

### Overview of the study Centre

The REVAMP study has been funded by the Indian Council of Medical Research (ICMR) as a Centre for Advanced Research Project - “Investigating mechanisms leading to preeclampsia” (5/7/1069/13-RCH dated 31-03-2017). The study is being carried out at the Interactive Research School for Health Affairs (IRSHA) in collaboration with Bharati Medical College and Hospital, Pune; Gupte Hospital and Research Centre, Pune; and Council of Scientific and Industrial Research-Centre for Cellular and Molecular Biology (CSIR-CCMB), Hyderabad. The current study is ongoing and the total duration of the study is five years i.e. from 31st March 2017 – 30th March 2022.

### Ethical considerations

The study has been approved by the Institutional Ethics Committee, Bharati Vidyapeeth Deemed University (IEC/2015/37, dated 03.10.2015).

### Approach to study population and informed consent

The ICMR Centre for Advanced Research Project was set up in March 2017. Patients coming to the outpatient departments of the Department of Obstetrics and Gynecology of Bharati Vidyapeeth Medical College and Hospital, Pune, and Gupte Hospital and Research Centre, Pune form the participants of the current study. Pregnant women attending the hospital for their first obstetric visit are informed about the study and their participation is solicited. Written consent is obtained from the women, including broad consent for further measurements of biochemical and molecular markers.

### Eligibility

#### Inclusion criteria

All women aged 18–45 years, with a singleton/twin pregnancy of gestation at 11–14 weeks desiring to continue the pregnancy and agreeing to deliver at the above hospitals are included in the study.

#### Exclusion criteria

Women are excluded if they have a history of cardiovascular disease (CVD), seizure disorder or liver disease. Women whose fetus is diagnosed to have a congenital abnormality at the 18–22 weeks scan are excluded, as are women with bleeding disorders, HIV infection or HBsAg.

#### Diagnosis of preeclampsia

As per the American College of Obstetrics and Gynecology (ACOG), 2013 guidelines, preeclampsia is diagnosed by the occurrence of new-onset hypertension along with new-onset proteinuria. Hypertension is defined as systolic blood pressure ≥ 140 mmHg and/or diastolic blood pressure ≥ 90 mmHg on at least two measurements taken at least 4 h apart. Proteinuria is defined as the excretion of 300 mg protein or higher in a 24 h urine specimen, or a protein/ creatinine ratio ≥ 0.3, or 1+ or higher on a dipstick test performed on two random urine samples collected at least 4 h apart. Preeclampsia, in the absence of proteinuria is now defined as the presence of hypertension along with any one of the following: thrombocytopenia, impaired liver function, development of renal insufficiency, pulmonary edema, or new-onset cerebral or visual disturbances [53].

Women who develop complications due to preeclampsia, including eclampsia, placental abruption, HELLP syndrome (hemolysis, elevated liver enzymes, low platelet count) and fetal death are also included. Gestational age will be based on the last menstrual period (LMP) date unless it differs from the gestation derived from the crown rump length at the first ultrasound scan (11–14 weeks) by >+/− 7 days, in which case the latter will be used. By using an LMP-based gestation rather than ultrasound-based gestation will enable us to detect differences in early fetal growth between case and control groups. Management of preeclampsia will be as per the clinician’s discretion and will be carefully documented.

### Sample size and power

Our earlier preliminary study on erythrocyte fatty acid levels in women with preeclampsia (*n* = 58) and normotensive controls (*n* = 137) at 16–18 weeks of gestation showed a mean difference of 0.3 and standard deviation of 0.8 [49]. We aim to study 100 women with preeclampsia and 200 normotensive women, which will give 87% power to detect a difference of the same magnitude when alpha is kept at 0.05. If the standard error is increased by 10%, the power will become 80%. For every case of preeclampsia, 2 normotensive controls will be randomly chosen from all non-preeclampsia pregnancies. Considering the incidence of preeclampsia to be around 8–10%, we will enroll ~ 1000 pregnant women until we get 100 cases of preeclampsia with complete data collection.

### Collection of information on individual study participants

All consenting participants are recruited at 11–14 weeks of gestation (visit 1 – V1), assigned a unique participant code, and subsequently followed across gestation at three time points viz. 18–22 weeks (visit 2 – V2), 26–28 weeks (visit 3 – V3) and at delivery. All data are recorded in a booklet that includes the various questionnaires, covering the following:

- ***Demographic characteristics including standard of living index (SLI):*** Data on the socioeconomic status of the participants is being collected using the standard of living index (SLI), a method developed by the International Institute for Population Sciences, Mumbai and used in India’s National Family Health Survey 2 [54]. Information of type of housing, land ownership, water and sanitation facilities, and ownership of household assets is used to derive a total score, with higher scores reflecting higher socioeconomic status.
- ***Participant’s personal and family medical history:*** A detailed interview is conducted to record the menstrual, obstetric (parity, gestation, mode of delivery), past and family history including information on maternal infection during pregnancy. Anthropometry (height and weight) of the pregnant women is recorded at all four time points.
- ***Clinical examination across gestation at four different time points:*** Table 1 gives a list of the various clinical measures being recorded. Oral glucose challenge test is carried out as per the Diabetes In Pregnancy Study Group of India (DIPSI) method [55].
- ***Physical activity:*** The women’s physical activity (1 month recall) is recorded across gestation at three different time points i.e. V1, V2 and V3 using a physical activity questionnaire categorized mainly in 3 main domains – occupation, travel and leisure activities. The questionnaire includes daily activities that were broadly categorized by intensity (light/moderate/heavy), type of household activities, and the total activity i.e. the total amount of time spent in hours per day (work, leisure, sleeping, travelling). A daily score is calculated where higher scores indicate more activity.
- ***Dietary assessments:*** Food frequency and 24-h recall questionnaires are administered by trained nutritionists. In addition, prescriptions for nutritional supplements such as iron, folate, calcium, zinc, and omega-3 fatty acids are documented.

i) The food frequency questionnaire (FFQ) (1 month recall) is administered across gestation at three different time points i.e. V1, V2 and V3 to estimate the frequency of consumption of different food items which are identified using the “Nutritive Values of Indian Foods” [56, 57]. The questionnaire consists of 17 food groups, each comprising ~ 10 food items: beverages; roti/Indian bread; rice; dal; whole pulses; vegetables; green leafy vegetables; papad; chutney/pickles; salad; foods consumed during fasting; fruits; non-vegetarian foods; milk and milk products; bakery products; fermented foods; foods consumed during festivals; type of oils and fast food items. Standardized utensils are used to record the quantity and the number of units of food items consumed. The frequency of intake of foods is then calculated and represented as monthly scores.
ii) The 24-h recall questionnaire is administered at the same time points and also includes assessment of portion size.

f) ***USG and Color Doppler assessments:*** USG and Color Doppler assessments are carried out at 11–14 weeks, 18–22 weeks and at 32–35 weeks of gestation (Table 2) and are in accordance with the International Society of Ultrasound in Obstetrics and Gynecology (ISUOG) and Fetal Medicine Foundation (FMF) protocols [58].

**Table 1:** Clinical examination across gestation at different time points

Time points	Clinical and Laboratory Investigations		
	Clinical examination	Blood examination	Urine routine test
11-14 weeks	• Systolic BP	• Hemogram	• Physical examination
	• Diastolic BP	• HBsAg	• Chemical examination
	• Pulse	• HIV	• Microscopic examination
	• Edema	• OGCT	
	• RS	• TSH	
	• CVS	• PAPP-A	
	• PA	• ß-HCG	
		• Creatinine	
18-22 weeks	• Systolic BP	• Hemogram	• Physical examination
	• Diastolic BP		• Chemical examination
	• Pulse		• Microscopic examination
	• Edema		
	• RS		
	• CVS		
	• PA		
26-28 weeks	• Systolic BP	• Hemogram	• Physical examination
	• Diastolic BP	• OGCT	• Chemical examination
	• Pulse	• TSH	• Microscopic examination
	• Edema		
	• RS		
	• CVS		
	• PA		
At delivery	• Systolic BP	• Hemogram	• Physical examination
	• Diastolic BP		• Chemical examination
	• Pulse		• Microscopic examination
	• Edema		
	• RS		
	• CVS		
	• PA		
Cord blood	-	• Hemogram	-

*BP* blood pressure, *CVS* cardiovascular system, *HBsAg* hepatitis B surface antigen, *HIV* human immunodeficiency virus, *OGCT* oral glucose challenge test [Diabetes In Pregnancy Study Group of India (DIPSI method)] [56], *PA* per abdomen, *PAPP-A* pregnancy-associated plasma protein A, *RS* respiratory system, *ß-HCG* beta human chorionic gonadotropin, *TSH* thyroid stimulating hormone

**Table 2:** Assessment of color Doppler and ultrasonography across gestation at different time points

Time points	Radiology Measurements	
	Ultrasonography (USG)	Color Doppler
11-14 weeks	• CRL	• Mean uterine artery PI
	• NT	• Ductus venosus
18-22 weeks	• BPD	• Mean uterine artery PI
	• HC	
	• AC	
	• FL	
	• Estimated fetal weight	
	• Placenta position	
	• Placenta previa	
	• Liquor quantity	
	• Anomaly	
32-35 weeks	• BPD	• Mean uterine artery PI and RI
	• HC	• Umbilical artery PI and RI
	• AC	• Fetal middle cerebral artery PI and RI
	• FL	
	• Estimated fetal weight	
	• Placenta position	
	• Placenta previa	
	• Liquor quantity	
	• Anomaly	
	• AFI	

*AC* abdominal circumference, *AFI* amniotic fluid index, *BPD* biparietal diameter, *CRL* crown rump length, *FL* femur length, *HC* head circumference, *NT* nuchal translucency, *PI* pulsatility index, *RI* resistance index, *USG* ultrasonography

### Collection of samples from individual study participants

#### Maternal and cord blood samples

Maternal blood is collected into vacutainers at four different time points (i.e. V1, V2, V3 and at delivery) for each participant. Similarly, cord blood is also collected at delivery. The blood is processed to separate serum, plasma, lymphocyte, and erythrocyte fractions which are stored at − 80 °C until further analysis. These fractions are aliquoted in a number of vials for prevention of freeze thaw cycles. An inventory (manual and computerized) is maintained for each sample stored in the freezer.

#### Placental tissue

**Fig. 3:**
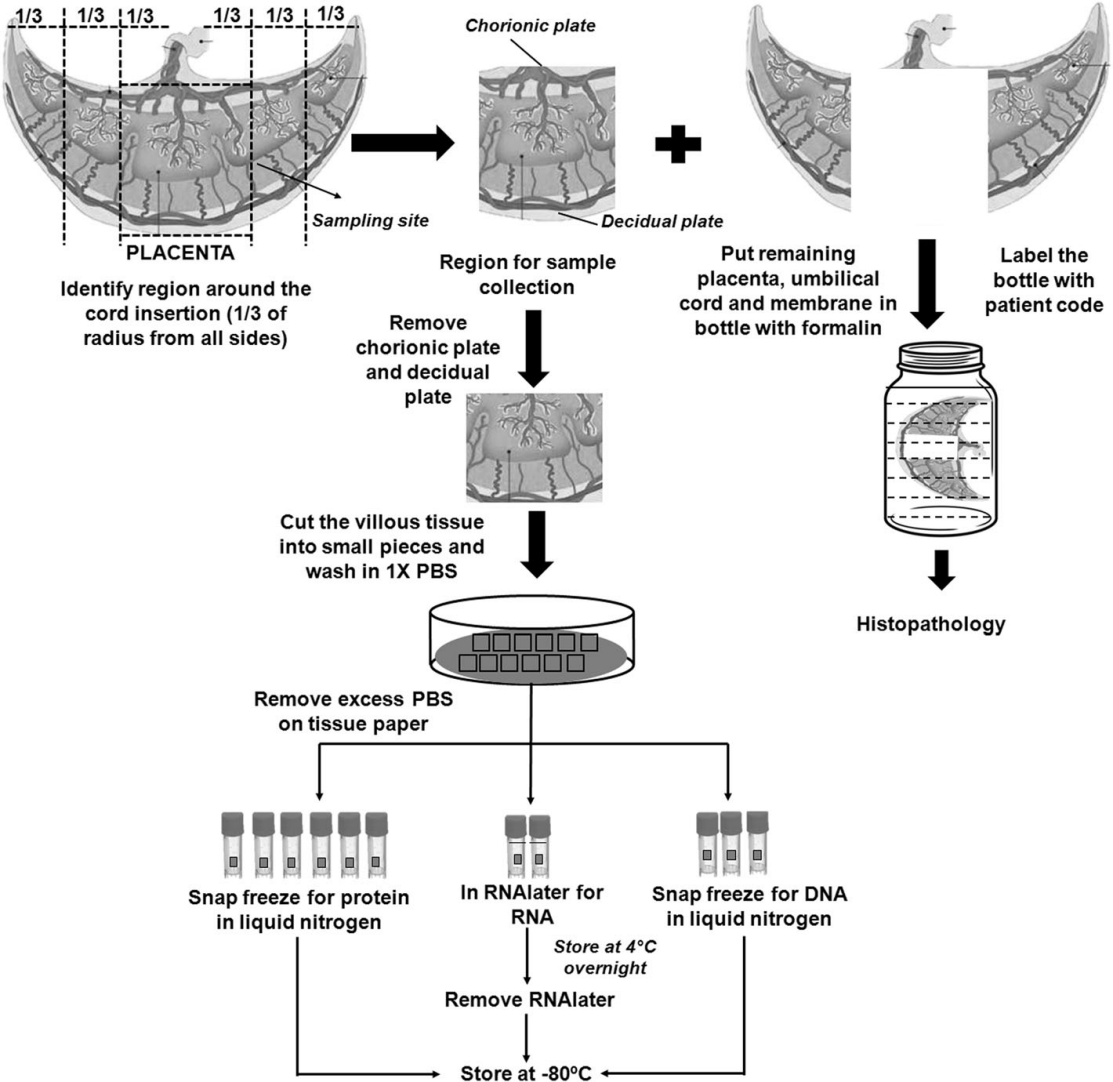
Placenta Collection and Processing. DNA, deoxyribonucleic acid; PBS, phosphate-buffered saline; RNA, ribonucleic acid

Figure 3 shows our methods for collection and processing of placental samples. Fresh placentae obtained from all pregnancies immediately after delivery are washed with working-strength solution of phosphate-buffered saline (1X PBS). The fetal membranes are trimmed across the placenta edge and the umbilical cord is cut 2 cm away from the insertion point. Different placental characteristics such as its shape and type of cord insertion, and measurements of the major axis, minor axis, breadth, thickness, and trimmed weight are recorded. The placenta is also checked for any gross abnormalities. The area around the cord insertion is considered as the region of interest and one-third radius area around all sides of the cord insertion is cut. The remaining placenta tissue, umbilical cord, and fetal membranes are put in formalin for histopathology. From the region of interest that is cut, the basal plate and the chorionic plate are cut off and discarded. Small tissue pieces are made from this region and rinsed in 1X PBS to remove maternal and fetal blood. These tissue pieces are then stored in cryovials containing RNA stabilization reagent (RNAlater) and the remaining tissue pieces are placed in cryovials and snap frozen in liquid nitrogen and then stored at − 80 °C until analyzed. An inventory (manual and computerized) is maintained for each placenta aliquot stored in the freezer.

### Laboratory measurements

**Fig. 4:**
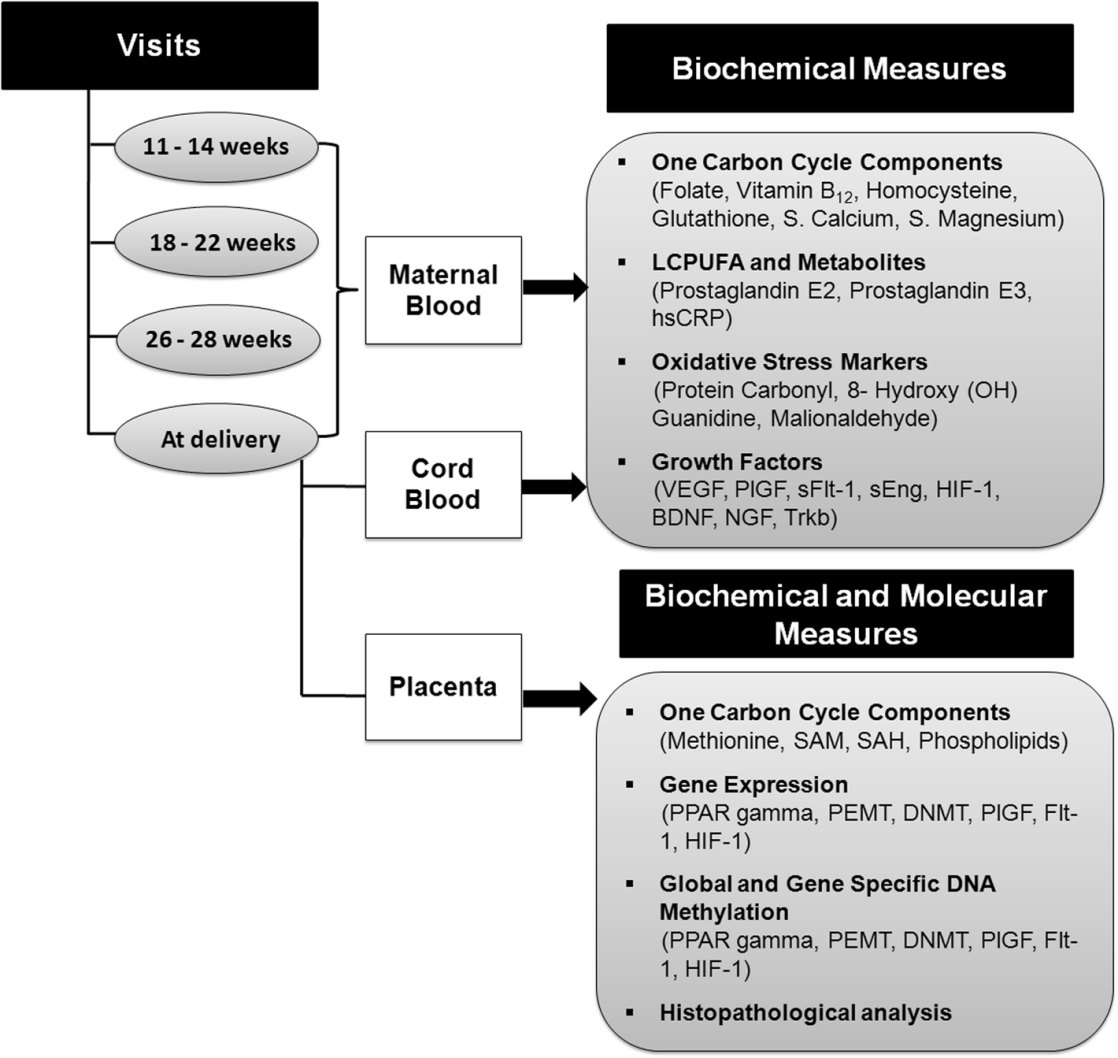
Estimation of various biochemical and molecular measures at different time points. BDNF: Brain-derived neurotrophic factor; DNMT, DNA methyl transferase; HIF-1: Hypoxia-inducible factor-1; hsCRP: high sensitivity C-reactive protein; NGF: nerve growth factor; PEMT, phosphatidylethanolamine methyl transferase; PlGF: placental growth factor; PPAR gamma: peroxisome proliferator-activated receptor gamma; SAH, S-adenosylhomocysteine; SAM, S-adenosylmethionine; sEng: soluble endoglin; sFlt-1: soluble fms-like tyrosine kinase-1; Trkb: tyrosine receptor kinase B; VEGF: vascular endothelial growth factor

Figure 4 shows the collection of maternal blood, cord blood, and placental samples at different time points. Various biochemical and molecular measures to be estimated are summarized in the Fig. 4. The biochemical measures and the molecular measures such as gene expression and global DNA methylation are carried out at IRSHA, Pune. Gene specific methylation analysis will be carried out CSIR-CCMB, Hyderabad. Histopathological analysis on placental samples will be carried out at Department of Pathology, Bharati Vidyapeeth Medical College, Pune.

### Biorepository

This project allows us to form a secure Indian Biobank consisting of the various biological samples collected under this project, and the associated data, and facilitate access to other investigators. External researchers can propose projects, and what the sample resource and/or data they require, and sharing will be carried out following approval by a scientific steering committee. The samples collected are stored at IRSHA, Bharati Vidyapeeth (Deemed to be University), Pune. Strict procedures are used to protect the confidentiality of the study participant’s data and samples. Memorandum of understanding (MOUs) will be undertaken as per Government of India rules and regulations.

Different aliquots for different fractions i.e. plasma, erythrocyte, lymphocytes, serum, and placenta are stored in different − 80 °C freezers. A stock record (manual and computerized) is maintained for each sample, including date of sample collection and processing, sample quantity, number of aliquots for each sample, position of each aliquot in the freezer, and end user details. Access to the freezer is restricted to authorized trained personnel only.

### Follow-up of the children

#### Anthropometry

Infant weight, length, chest and head circumference are measured at birth and repeated at time points to match the vaccination routine/schedule: 6, 10, and 14 weeks, and 6, 9, 12, 15, 18, and 24 months. These measurements are made by trained staff according to standardized protocols.

#### Developmental assessments in children

At 2 years of age, the Development Assessment Scale for Indian Infants (DASII) will be administered [59]. All tests will be carried out in the local language, in a single session of 60–90 min in the morning, in a quiet room, by a trained master’s level child psychologist certified in DASII who will be unaware of the mother’s pregnancy status. Psychologists will administer the scales in consultation with an experienced Developmental Pediatrician.

### Data quality assurance

The laboratory staff will be blinded to the identity of the samples. Inter- and intra- observer/laboratory reliability assays have been performed. For example, inter-laboratory comparison for angiogenic factors was undertaken at All India Institute of Medical Sciences (AIIMS), Delhi and at IRSHA. An inter-laboratory comparison for fatty acid analysis by Gas Chromatography was carried out at three laboratories – IRSHA, AIIMS, Delhi and National Institute of Nutrition (NIN), Hyderabad. Color Doppler and USG assessments are validated on a sub-sample by an expert in the field.

All study participants’ information is stored in locked filing cabinets with limited access. Double data entry is performed to reduce errors. Data confidentiality is maintained and datasets are anonymized before any analysis. An experienced statistical consultant will help in selecting and implementing data analysis methods.

### Statistical analysis

#### Data processing

Skewed variables will be transformed to normality. Dietary data from the food frequency questionnaires and 24-h recalls will be processed to derive intakes [frequencies and amounts (gram weights)] of specific foods (e.g. pulses/legumes, meat/fish, green leafy vegetables), dietary patterns derived using Principal Components Analysis, and nutrient intakes calculated using food content tables.

#### Predictors of preeclampsia

Comparisons of early pregnancy diet variables, blood fatty acids and one carbon nutrients and other biomarkers will be made between cases (women who develop preeclampsia) and controls (women without preeclampsia selected randomly from all other births during the study), 2 controls per case. Multiple logistic regression analysis will be used to examine associations of the early pregnancy measures with preeclampsia, adjusting for potential confounders such as maternal age, parity, anthropometry, socio-economic status and gestational diabetes mellitus (GDM). All associations will be examined for differences in effect between categories of maternal body mass index (BMI) and height, between male and female fetuses/newborns, and between GDM and non-GDM pregnancies, using stratified analyzes and interaction tests. We will explore the proposed mechanistic pathway/causal chain depicted in Fig. 2, using multiple regression initially, and other methods such as structured equation modelling. We will adjust significance estimates for multiple testing where appropriate.

We will explore the potential clinical utility of the early pregnancy measures as predictors of preeclampsia, using specificity, sensitivity, and positive and negative predictive values. We will also examine the proportion of preeclampsia attributable to these measurements and combinations of measurements.

#### Predictors of fetal and childhood size and growth

We will use multiple linear regression to analyze the relative contributions of clinical, nutritional, and biochemical/molecular parameters to variability in fetal size at each scan, fetal growth between scans, and newborn weight and length, taking into account gestational age, fetal/newborn sex and maternal characteristics.

#### Preeclampsia and early pregnancy biomarkers as predictors of child outcomes

We will use multiple linear and logistic regression to assess differences between the preeclampsia and control groups in outcomes measured in the children up to the age of 2 years. The outcomes will include infant weight and height, derived SD scores for weight and height based on World Health Organization (WHO) reference data, categories of nutritional status (stunting, normal weight, underweight, overweight and wasting), growth trajectories from birth to 2 years and DASII developmental scores at age 2 years. Adjustment will be made for the child’s age and sex, gestational age at birth, and maternal age, BMI, and socioeconomic status. We will also explore associations of maternal nutritional and biochemical measures in early pregnancy and of more ‘distal’ components of the proposed mechanistic pathway such as maternal and cord blood growth factor levels, with the child outcomes.

#### Analysis of the epigenetic data

The CpG methylation data will be generated by pyrosequencing on the targeted regions of various genes in placentae from women with preeclampsia and normotensive women. Descriptives for cases and controls shall be reported followed by regression analysis for assessing any significant differences. The technical and biological covariates shall be correlated with methylation percentage at each CpG and significantly correlated covariates shall be adjusted in regression models. CpG methylation clustering will also be analyzed region-wise.

### Governance/ meetings

To initiate the project a series of meetings were held with the clinicians from both the participating hospitals. Subsequent meetings were held with clinicians to finalize various aspects related to sample collection and processing. Discussions were also held with the collaborators from CCMB to finalize issues related to sample processing for epigenetic work. Aspects related to information on participants’ family and personal history, SLI, physical activity, FFQ and 24 h dietary recall data, infant proforma, feeding practices, and child follow-up data were discussed and finalized with expert advisors.

A Scientific Advisory Committee has been formed which consists of clinicians, research scientists, and research experts viz. Professor Caroline HD Fall (University of Southampton, UK), Professor C. S. Yajnik (KEM Hospital, Pune), Dr. Anura Kurpad (St. John’s Medical College, Bangalore), Dr. K. Kumaran (MRC Lifecourse Epidemiology Unit, Southampton & Holdsworth Memorial Hospital, Mysore), Dr. Julian Crasta (St. John’s Medical College, Bangalore), Dr. Lakshmy Ramakrishnan (All India Institute of Medical Sciences, New Delhi), Dr. Arun Kinare (Bharati Medical College and Hospital, Pune), Dr. Suhas Otiv (KEM Hospital, Pune). A series of annual meetings are being held with the advisory committee to discuss scientific aspects and the monitoring of the progress made in this project. Reports are submitted to Indian Council of Medical Research (ICMR) and a face-to-face meeting is held each year.

## Discussion

### Translational potential of the study

The present study will be useful in:

- ***Development/validation of biomarkers for early prediction of preeclampsia:*** There is a need for the development of biomarkers which would help in the early diagnosis and treatment of preeclampsia. This is important as treatments are most likely to be effective if started in early gestation. A combination of clinical information along with blood biomarkers would prove to be more realistic and beneficial than a single marker as a predictive tool for preeclampsia.
- ***Providing information on mechanistic aspects of preeclampsia:*** By examining both biochemical and molecular mechanisms, this study may lead to a more complete understanding of the etiology of preeclampsia.
- ***Understanding the neurodevelopmental outcome of children born to women with preeclampsia:*** It is well known that the offspring born to mothers with preeclampsia are at increased risk for developing neurodevelopment disorders and are associated with impaired cognition. This study will help to better understand the maternal and cord biochemical and molecular mechanisms associated with developmental scores in children born to mothers with preeclampsia.
- ***Correlation of USG and color Doppler findings and histopathological examination of placenta:*** This study will help understand the correlation of USG and color Doppler findings with the histology of placenta as well as maternal biochemical parameters. This may have high predictive value to identify women at risk for developing preeclampsia.
- ***Establishing a biorepository:*** The establishment of a biorepository will provide an invaluable resource to be shared with other investigators for future research on other factors influencing adverse pregnancy outcomes.

### The relevance of the proposed study

Children born to mothers with preeclampsia are at an increased risk of developing cardiovascular disease, diabetes and neurodevelopmental disorders in later life. The proposed study will systematically and comprehensively investigate the key biochemical and molecular mechanisms contributing to preeclampsia. We expect to identify novel circulating proteins which predict the risk of developing preeclampsia. Identification of such factors may provide clues for therapeutic interventions. The study will furthermore give insights into novel epigenetic mechanisms in the placenta which may lead to impaired placentation and fetal growth in preeclampsia. This study will also help us to establish the correct reference baseline for vital nutrients like omega-3 fatty acids for the Indian population as, such data is long overdue. An improved understanding of these factors will help guide development of public health interventions to reduce and curtail the burden of non-communicable diseases (NCDs) in India.

### The outcome of proposed study

The proposed comprehensive basic science, translational and public health oriented collaborative research plan between basic scientists and clinicians will help to understand the origin of preeclampsia in the mother and its metabolic consequences in their offspring. This research includes identifying biomarkers like growth factors in the plasma for the early prediction of risk for developing preeclampsia.

## Data Availability

“Not applicable”.

## Abbreviations

AA: Arachidonic acid
ACOG: American College of Obstetrics and Gynecology
AIIMS: All India Institute of Medical Sciences
ALA: Alpha linolenic acid
BDNF: Brain derived neurotrophic factor
BMI: Body mass index
CCMB: Centre for Cellular and Molecular Biology
CSIR: Council of Scientific and Industrial Research
CVD: Cardiovascular disease
DASII: Development Assessment Scale for Indian Infants
DHA: Docosahexaenoic acid
DIPSI: Diabetes In Pregnancy Study Group of India
DNMT: DNA methyl transferase
FFQ: Food frequency questionnaire
Flt-1: fms-like tyrosine kinase-1
FMF: Fetal Medicine Foundation
GDM: Gestational diabetes mellitus
HELLP: Hemolysis, elevated liver enzymes, low platelet count
HIF-1: Hypoxia inducible factor-1
ICMR: Indian Council of Medical Research
IRSHA: Interactive Research School for Health Affairs
ISUOG: International Society of Ultrasound in Obstetrics and Gynecology
LCPUFA: Long chain polyunsaturated fatty acids
LMP: Last menstrual period
MDA: Malondialdehyde
MOU: Memorandum of understanding
NCD: Non-communicable diseases
NGF: Nerve growth factor
NIN: National Institute of Nutrition
PBS: Phosphate-buffered saline
PC: Phosphatidylcholine
PE: Phosphatidylethanolamine
PEMT: Phosphatidylethanolamine-N-methyltransferase
PlGF: Placental growth factor
PPAR: Peroxisome proliferator-activated receptor
REVAMP: Research Exploring Various Aspects and Mechanisms in Preeclampsia
SAH: S-adenosylhomocysteine
SAM: S-adenosylmethionine
sEng: Soluble endoglin
sFlt-1: Soluble Flt-1
SLI: Standard of living index
USG: Ultrasonography
VEGF: Vascular endothelial growth factor
WHO: World Health Organization
